# Towards standardization of next-generation sequencing of FFPE samples for clinical oncology: intrinsic obstacles and possible solutions

**DOI:** 10.1186/s12967-017-1125-8

**Published:** 2017-01-31

**Authors:** Maxim Ivanov, Konstantin Laktionov, Valery Breder, Polina Chernenko, Ekaterina Novikova, Ekaterina Telysheva, Sergey Musienko, Ancha Baranova, Vladislav Mileyko

**Affiliations:** 1 Moscow Institute of Physics and Technology (State University), Dolgoprudny, Moscow Region, 141700 Russia; 2 Atlas Biomed Group, Moscow, 121069 Russia; 3 Institute of Chemical Biology and Fundamental Medicine of SB RAS, Novosibirsk, 630090 Russia; 4 N.N. Blokhin Russian Cancer Research Center, Ministry of Health of the Russian Federation, Kashirskoe sh. 24, Moscow, 115478 Russia; 5 Federal State Budgetary Institution Russian Scientific Center of Roentgenoradiology (RSCRR) of the Ministry of Healthcare of the Russian Federation (Russian Scientific Center of Roentgenoradiology), Moscow, 117485 Russia; 6 Research Centre for Medical Genetics, Moscow, 115478 Russia; 7 Center for the Study of Chronic Metabolic and Rare Diseases, School of System Biology, George Mason University, Fairfax, VA USA

**Keywords:** NGS, Non-small cell lung cancer, Cancer, Oncology, Precision oncology, Targeted therapy

## Abstract

**Background:**

Next generation sequencing has a potential to revolutionize the management of cancer patients within the framework of precision oncology. Nevertheless, lack of standardization decelerated entering of the technology into the clinical testing space. Here we dissected a number of common problems of NGS diagnostics in oncology and introduced ways they can be resolved.

**Methods:**

DNA was extracted from 26 formalin fixed paraffin embedded (FFPE) specimens and processed with the TrueSeq Amplicon Cancer Panel (Illumina Inc, San Diego, California) targeting 48 cancer-related genes and sequenced in single run. Sequencing data were comparatively analyzed by several bioinformatics pipelines.

**Results:**

Libraries yielded sufficient coverage to detect even low prevalent mutations. We found that the number of FFPE sequence artifacts significantly correlates with pre-normalization concentration of libraries (rank correlation −0.81; p < 1e−10), thus, contributing to sample-specific variant detection cut-offs. Surprisingly, extensive validation of EGFR mutation calls by a combination of aligners and variant callers resulted in identification of two false negatives and one false positive that were due to complexity of underlying genomic change, confirmed by Sanger sequencing. Additionally, the study of the non-EGFR amplicons revealed 33 confirmed unique mutations in 17 genes, with TP53 being the most frequently mutated. Clinical relevance of these finding is discussed.

**Conclusions:**

Reporting of entire mutational spectrum revealed by targeted sequencing is questionable, at least until the clinically-driven guidelines on reporting of somatic mutations are established. The standardization of sequencing protocols, especially their data analysis components, requires assay-, disease-, and, in many cases, even sample-specific customization that could be performed only in cooperation with clinicians.

## Background

The advent of next-generation sequencing and other genomic technologies makes it routinely possible today to investigate DNA specimens extracted from human tumors for gene mutations, chromosomal aberrations, differential expression of mRNA and epigenetic alterations. Such detected molecular changes are extensively used for integrative analyses aimed at the identification of key dysregulated pathways and importantly, the establishment of molecular classifiers indispensable for targeted therapy and personalized management of malignant disorders [1].

Due to relatively high prevalence of the mutations in the epidermal growth factor receptor (EGFR) that has led to the development of EGFR tyrosine kinase inhibitors (EGFR-TKIs), non-small cell lung cancer (NSCLC) has become a proving ground for the development of novel approaches for molecular typing [2]. Unfortunately, it has been established that EGFR-responsive patients with activating EGFR mutations eventually develop resistance to the treatment and progress either due to commonly acquired T790M mutations or to a variety of other molecular changes [3]. On the other hand, initial success in exploiting EGFR-TKI sensitivity paved the way for the development of other small molecular inhibitors as well as widespread introduction of the mutation analyses into the molecular subtyping of other tumors. Examples of actionable mutations include RAS (KRAS and NRAS), BRAF and PI3 K in colorectal cancer [4], BRAF or NRAS as well as KIT in malignant melanoma [5], and others. These and other genetic alterations continue to gain importance in companion diagnostics associated with recently marketed targeted therapies and investigational drugs [6–8]. As a consequence of efforts invested in this area, the number of clinically actionable variations that contribute to sensitivity or resistance of tumors to each of these drugs is dramatically expanding alongside available therapeutic solutions, especially in cases of metastatic and refractory diseases. Moreover, the numerous case reports demonstrating clinical response to the drugs prescribed off-label based on matching molecular evidence ensure the spread of a molecular-informed therapeutic decision-concept into oncologist office routine [9–12].

This avalanche of changes was precipitated by the replacement of conventional real-time polymerase chain reaction (PCR) or direct sequencing by Sanger with Next Generation Sequencing (NGS) which has recently revolutionized the field of molecular diagnostics. In addition to higher sensitivity and remarkable throughput, NGS allows gaining non-traditional kinds of information about extracted DNA specimen, including the prevalence of shorter fragments [13] and the degree of its sequence heterogeneity within each locus [14]. Nevertheless, a majority of NGS applications are currently limited to research use only, while its implementation in clinical practice awaits rigorous validation requiring establishing Clinical Laboratory Improvement Amendments (CLIA) and College of American Pathologists (CAP)-compliant performance characteristics. Thus, rapid entering of NGS into the space of clinical diagnostics is complicated by a number of loosely defined unknowns, including a coverage required for confident detection, data quality control, benchmarking bioinformatics pipelines currently producing discordant variant calls and overall validation of the robustness of NGS techniques.

Besides the technical obstacles outlined above, there are basic issues with existing “gold standard”: a number of novel, potentially very important insights into the tumorigenesis were gained by massive parallel sequencing for the first time, as they originated from observations that cannot be performed earlier due to intrinsic shortcomings of Sanger sequencing and conventional PCR. As example we can point to the clinical significance of intratumor heterogeneity with therapy resistance mutations being present with low prevalence already at the pretreatment assessment [15]. Currently, there is no consensus on how to differentiate these mutations from the FFPE artifacts [16]. Another set of important issues are incidental findings that may be revealed by NGS reads even if they were targeted for detecting of the mutation spectrum defined beforehand [17]. Examples of such incidental findings may include novel, not yet characterize mutations within target gene, common variants that may influence risks of other diseases or even germline mutations [18]. Such findings are generally hard to interpret in terms of their clinical relevance and lack a consensus on whether they should be communicated to physician or the patient at all. Finally, NGS allows one to detect mutations, which remain unseen if not being targeted by analysis. For instance, owing to implementation of the NGS into clinical practice, EGFR kinase domain duplication mutation has recently emerged as novel EGFR TKI sensitizing variant [19].

Despite recent publications of a number of clinical NGS guidelines clinical test development by a variety of organizations including American College of Medical Genetics [20] and College of American Pathologist [21], overcoming the obstacles mentioned above remains challenging, especially when the detected mutations are somatic, as the majority of guidelines are established only for the reporting of germline mutations. It is likely that both general recommendations and relevant protocols for sequencing, data analysis and clinical interpretation will continue to evolve in the process of sharing the outcomes of the tailoring of commercially available tests to the needs of particular practice or country, or panel customization. In this paper we describe particular obstacles we encountered while analyzing the clinical NGS dataset obtained using the TruSeq Amplicon—Cancer Panel (TSACP) and possible solutions to the problems presented.

## Methods

### Sample collection

Thirteen archived clinical tumor specimens from twelve lung cancer patients treated at Blokhin Russian Cancer Research Centre (RCRC) in 2014–2015 were randomly selected from respective existing registry. Another set of 13 samples was retrospectively randomly selected from a collection of Russian Scientific Center of Roentgenology and Radiology (RSCRR). Specimens of the latter set had already been screened for the presence of EGFR mutations; hence, this RSCRR set of samples was enriched with EGFR positive patients by design. Overall, we studied 26 tumor specimens including lung adenocarcinoma (n = 11, 42%), squamous cell carcinoma (n = 11, 42%) and the tumors of mixed or unknown histology (n = 4, 16%). The study protocol was approved by Atlas Biomed Internal Review Board. Written informed consent was provided by all patients at inception of the study, all analyses were based on archival data and stored in database with no connections to the patient identifiers.

### DNA extraction and sample quality control

Genomic DNA was extracted from formalin fixed, paraffin embedded (FFPE) tissues with the QIAamp DNA FFPE kit (Qiagen, USA) according to the manufacturer’s instructions and was eluted in a 25 μL volume. The extracted DNA specimens were further quantified using the Qubit dsDNA HS assay kit (Life Technologies/Fisher Scientific, USA) and samples with DNA concentration lower 50 ng/μl were concentrated to this value. DNA quality and quantity were further assessed using the Illumina FFPE QC Kit according to the manufacturer’s instructions.

### Library preparation and quality control

Sequencing libraries were prepared with the TSACP (Illumina, San Diego, USA), according to manufacturer’s protocol. Briefly, an oligo pool was hybridized to each genomic DNA sample. Following the removal of unbound oligos, target regions of interest flanked by sequences required for amplification were generated by extension and ligation and libraries were further PCR amplified. Library quality was assessed on a 2100 Bioanalyzer (Agilent Technologies, Santa Clara, California). Prior sequencing, the libraries were normalized following the manufacturer protocol and equal volumes were pooled to generate the final sequencing library. Primer panel designed to generate 212 amplicons within 48 cancer-related genes: *ABL1, AKT1, ALK, APC, ATM, BRAF, CDH1, CDKN2A, CSF1R, CTNNB1, EGFR, ERBB2, ERBB4, FBXW7, FGFR1, FGFR2, FGFR3, FLT3, GNA11, GNAQ, GNAS, HNF1A, HRAS, IDH1, JAK2, JAK3, KDR, KIT, KRAS, MET, MLH1, MPL, NOTCH1, NPM1, NRAS, PDGFRA, PIK3CA, PTEN, PTPN11, RB1, RET, SMAD4, SMARCB1, SMO, SRC, STK11, TP53*, and *VHL*.

### Sequencing and data analysis

Pooled libraries were sequenced using MiSeqDx (llumina) with a 2 × 150 paired-end sequencing design. Image processing and fastq file generation were further performed with CASAVA version 1.8.2 and RTA version 1.17.28 (Illumina). Reads were preprocessed for a removal of low-quality and too short nucleotide sequences using the Prinseq-lite program [22]. Minimum mean read quality score was set to Q30, and minimum length to 75 base pairs. Remaining paired-end reads were mapped to the GRCh37.p13 human genome employing Bowtie2 [23] software with varying parameters. After alignment, an exclusion of primers was performed employing in-house software. SAMtools (version 1.2) [24] was applied for the calling of germline variants, while somatic mutations were identified by Strelka (version 1.014) [25], Varscan (version 2.3.9) [26], ScalPel (version 0.5.3) [27] and Illumina Somatic Variant Caller (SVC) (version 3.1.6.4). GATK version 3.6 [28] was additionally used for indel realignment and other analysis. Germline polymorphisms were discriminated from the somatic based on their frequencies in human populations and presence in dbSNP [29] and COSMIC [30] databases. Recurrent artifact variant calls were discarded from the analysis employing in-house software. Protein variant annotation was performed using ANNOVAR [31], with non-coding or synonymous mutations discarded from further analysis. Copy number variations were detected using CNVPanelizer [32].

### Mutation verification

Following NGS sequencing, EGFR and KRAS (including codons 12, 13) mutations in samples from the RCRC set as well as KRAS mutations from RSCRR set were validated either by Sanger sequencing or Real-Time PCR. Samples from RSCRR set were pre-screened for EGFR mutations and, thus, not required further validation with orthogonal methods. EGFR mutations (including exons 18–21) were examined by Sanger Sequencing. Primer pairs were 5′-CTGAGGTGACCCTTGTCTCTG-3′ and 5′-CCAAACACTCAGTGAAAC-3′, 5′-TGCCAGTTAACGTCTTCCTT-3′ and 5′-CAGGGTCTAGAGCAGAGCAG-3′, 5′-CATTCATGCGTCTTCACCTG-3′ and 5′-TTATCTCCCCTCCCCGTATC-3′, and 5′-TGATCTGTCCCTCACAGCAG-3′ and 5′-GGCTGACCTAAAGCCACCTC-3′ for exons 18, 19, 20 and 21 respectively. Thermal cycling conditions included 5 min at 95 °C followed by 35 cycles of 95 °C for 30 s, 60 °C for 30 s, 72 °C for 1 min and one cycle of 72 °C for 7 min. The PCR products were further purified with USB ExoSapit (GE Healthcare, Uppsala, Sweden) followed by cycle sequencing with the BigDye Terminator version 3.1 cycle sequencing kit (Applied Biosystems) according to the manufacturer’s protocol and resolved on an ABI 3500xL sequencer (Applied Biosystems). Sequence chromatograms were analyzed by Sequencher software (Gene Codes Corp, Ann Arbor, MI), followed by manual review. KRAS codon 12, 13 mutations were examined by pyrosequencing as described previously [33].

## Results

### NGS sequencing results

We performed high-throughput sequencing employing amplicon library construction with primer panel targeting 212 regions of 48 oncogenes and tumor suppressor genes in 26 FFPE tumor samples. Approximately 91% of all reads from the Illumina MiSeq sequencer were successfully mapped to the reference genome, with the coverage depth of 720× for 82% of the target bases and 220× for 93% of the target bases. Across the samples, the overall mean coverage was 2084× and median—2016×. The frequency of amplicon drop-out was at 0.5%. These results indicate that a coverage resolution was high enough to identify somatic point mutations and short indels as well as the copy number variations.

In contrast to high coverage observed across entire panel, one or more amplicons within the genes *CDKN2A*, *FGFR3, GNA11, HRAS, MPL, SRC, STK11, VHL* and *SMO* displayed relatively low depth of the coverage in substantial proportion of DNA specimens. For many samples, amplicon-specific coverages were seen to drop as low as 140×. An extremely low coverage at less than 4% of the average amounts of reads across all amplicons and samples with the mean of 51× was detected in MPL gene, which, to the best of our knowledge, was not previously noted as deviating from average. Additionally, the mining of existing literature highlights that the sequencing of some regions within *RB1, HNF1A, NOTCH1* and *RET* might also result in consistently low coverage [34, 35]. In our study, uniformly high coverage of these genes was observed across all samples. It is possible that amplicon-specific coverage may vary form run to run, or depend on the batch of the primers or the library kit, or clinic-specific protocol of FFPE preparation.

For the mutations informing the treatment strategy specifically in the lung cancer, the coverage was consistently high, with the lower border of the 99.9% CI of coverage depth 1.5 times higher than the average across all the genes comprising TCASP panel. This quality of the coverage allowed us to proceed with other types of analysis aimed at highlighting problematic aspects of NGS testing in clinical oncology.

### DNA degradation artifacts may limit the accuracy of the test

The quantities and the quality of DNA extracted from tumor biopsies might vary from sample to sample. In particular, the tissue fixation process might result in extensive DNA degradation and, therefore, to introduction of artifactual bases. According to previous observations made using FFPE samples, false positive findings are predominantly represented by C:G > T:A base substitutions with frequencies in range of 0–10% of the covering reads [36, 37]. Typical assessment by Sanger sequencing is insensitive to the variants present at frequencies lower than 20% [38]. In contrast, the diagnostic with NGS overcomes this obstacle by increase in the coverage proportional to the rarity of the variant. Given that clinical guidelines recommend detection of the variants that are present in 5 or 2.5% of the covering reads for mutations associated with the response and the resistance, respectively, poses the problem of discrimination between verifiable mutations and artifacts of the FFPE processing.

**Fig. 1:**
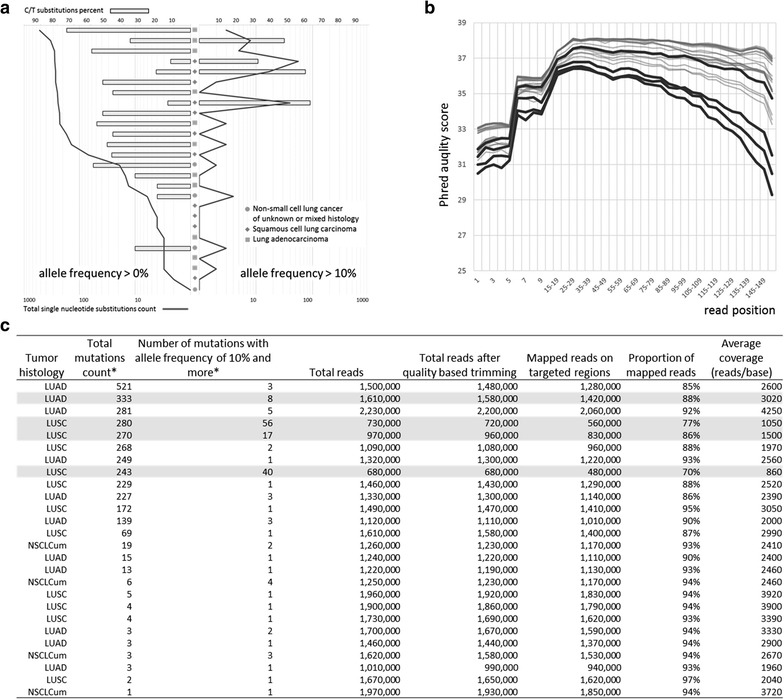
The description of potential artifacts detected in FFPE samples. **a** Mutation counts (*solid* *line*) and C/T base substitution rates (*bar histogram*) at two allele frequency ranges across all samples were sorted according to the total mutation counts for given specimen. There was a trend of increasing C/T substitution rate along with total mutation count. Four samples did not follow this trend. Only these samples and none of the others showed very high mutation counts at allele frequencies of more than 10% with the accordingly high C/T substitution rate in this range. Pre-normalization library concentrations were low for all four samples in question. **b** Per-base Phred quality scores for each position across all samples. Four samples with high mutation counts at allele frequencies of more than 10% are highlighted by *bold lines*. **c** Overall sequencing results for all samples. Four samples of interest are respectively highlighted

Across all samples studied, we identified a bit over 3300 mutations with the mean C > T base substitution proportion of 44%. Among these, approximately 3200 mutations were detected with the allele frequency ranges of 0–10%. Yet, four out of 26 FFPE specimens displayed high mutation rate with average allele frequencies of more than 10%, with eight, seventeen, forty and fifty-six of these highly prevalent mutations detected in same DNA sample, respectively (Fig. 1a). Importantly, a majority of these highly frequent mutations were C > T substitutions. In four specimens of interest, they were at 34, 50, 63 and 65%, respectively. In each sample with aberrant count of high prevalent substitution, prevalence of the C > T substitutions in allele frequency range from 0 to 10% tended to be lower as compared with the other samples (with *p* value ranging from 0.015 to 2e−7), thus, demonstrating a peculiar trend.

In addition, for three out of four samples with high mutation loads, the coverage was significantly reduced due to low total read counts (p value ranging from 0.026 to 5e−3) (Fig. 1c). Similarly, the percentages of reads passing base quality filters for these three samples were also smaller (Fig. 1b). For one out of four samples with high mutation loads, the coverage was trending along with the majority of samples (p-value = 0.61).

**Fig. 2:**
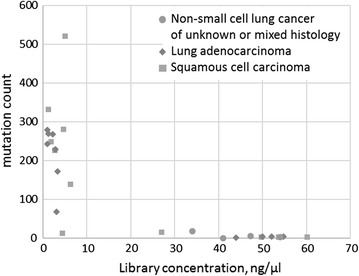
The mutations counts correlate with the pre-normalization library concentrations. Samples with highest pre-normalization library concentrations were found to harbor fewer mutations, most probably due to lower amounts of DNA degradation artifacts, while samples with lowest pre-normalization library concentrations harbored a diverse spectrum of the detectable variants (Spearman’s rank correlation −0.81; p < 1e^−10^)

An insight on initial DNA quality may be derived by assessing sequencing library concentrations before the normalization. In specimens of interest, these concentrations were at 1.0, 1.0, 1.3 and 1.3 ng/μl, while for the rest the lowest sequencing library concentration was at 1.9 ng/μl (p < 1e−5, by Wilcoxon-Mann–Whitney Test). Furthermore, the DNA specimens converted to the libraries with high pre-normalization concentrations displayed lowest total mutation counts (Fig. 2). It seems that pre-normalization concentrations of libraries are indicative of the degree of DNA degradation in a given specimen and could be useful for calculations of sample-specific expected rates of artifactual findings.

### DNA specimens may yield multiple somatic mutations associated with potentially important incidental findings

Owing to advances in the development of TKIs that target EGFR, the screening for point mutations within EGFR already became an integral part of routine examination of lung tumor specimens [39]. However, recent studies indicated that the mutations in the genes of MAPK and PI3K-AKT pathways may influence the response to EGFR TKI and, therefore, are useful as both predictive and prognostic markers. These observations prompted us to perform retrospective validation of the presence of NGS-detected somatic variations in *PIK3CA, BRAF, KRAS, NRAS, HER2, ALK* and *MET* genes employing Sanger sequencing or quantitative Real-Time PCR.

In a study of 26 FFPE tumor specimens, activating *EGFR* mutations associated with sensitivity to the EGFR TKI therapy were detected in eight cases. Among these, three tumor specimens were found to harbor coding frame point mutation p.L858R (or p.Leu858Arg), four tumors had the deletion of exon 19 and a single sample had an insertion of exon 19. The EGFR TKI resistance associated mutation p.T790M (or p.Thr790Met) was detected in a single sample with p.L8585R variant of *EGFR*.

As TSACP panel covers 48 oncogenes and tumor suppressor genes, we were able to extend the analysis of 26 FFPE lung cancer specimens beyond *EGFR* gene. Assuming that non-EGFR mutations gain their therapeutic significance only when present in a relatively major clone, the allele frequency cutoffs were redefined at 10%. After filtering out all non-recurrent mutations detected exclusively in samples with low pre-normalization library concentrations, we identified 33 unique non-EGFR point mutations including 25 single nucleotide variations and 8 small insertions/deletions (Table 1). Of these, 28 variants were likely of somatic origin, and further 5 were likely germline. In each particular case, the determination was made after taking into account the presence of the mutation in dbSNP and COSMIC databases, and the reported prevalence in human populations or in cancer specimens. The highest rate of somatic mutations was observed in *TP53* gene with eleven alterations detected. Overall, previously described non-EGFR mutations known to modify the response to drug therapy were identified in 8 patients, and including the mutations in *TP53, KRAS, PIK3CA, HRAS, AKT1* and *CTNNB1*. Of particular interest, the mutations of KRAS were detected in 5 samples including one sample with simultaneous presence of two KRAS mutations (p.Gly12Cys and p.Ala146Thr), though only two of the KRAS positive samples harbored high prevalent mutations, while the rest, thus, could not be validated with orthogonal methods. Additionally, *PIK3CA* mutation p.Glu545Lys was detected in single case.

**Table 1:** Identified exonic mutations in non-EGFR genes

Gene	Protein sequence variation	Patient	Tumor histology	Mutant allele frequency (%)	Variant impact
HNF1A	p.Gly306 fs	5	LUAD	15	Deleterious
TP53	p.Gly272 fs	9	LUAD	15	Deleterious
TP53	p.Val173Met	10	LUAD	17	Deleterious [40]
MLH1	p.Ser406Asn	12	LUAD	15	Deleterious [41]
KIT	p.Glu76Asp	12	LUAD	50	Unknown
TP53	p.Leu206_Arg209del	12	LUAD	70	Unknown
ABL1	p.Thr243Ile	65	LUAD	15	Unknown
KRAS	p.Gly12Cys	65	LUAD	31	Activated
CTNNB1	p.Ser33Phe	89	LUAD	11	Activated [42]
KRAS	p.Gly12Asp	90	LUAD	30	Activated
TP53	p.Cys238Tyr	91	LUSC	44	Deleterious [40]
NOTCH1	p.Leu1600Pro	105	LUSC	11	Activated [43]
TP53	p.Arg175 fs	106	LUSC	56	Deleterious
HRAS	p.Gly13Val	120	LUSC	82	Activated [44]
AKT1	p.Glu17Lys	120	LUSC	4	Activated [45]
TP53	p.Ser215Gly	131	LUSC	45	Deleterious [40]
ATM	p.Asn856Ile	140	LUSC	10	Unknown
TP53	p.His214Arg	150	LUSC	33	Deleterious [40]
TP53	p.Arg337Pro	152	LUSC	50	Deleterious [40]
VHL	p.Lys171Arg	161	NSCLC	18	Deleterious [46, 47]
TP53	p.Arg248Gln	161	NSCLC	31	Deleterious [48, 49]
TP53	p.Ser185 fs	187	NSCLC	17	Deleterious
RB1	p.Ser576 fs	187	NSCLC	18	Deleterious
PIK3CA	p.Glu545Lys	193	NSCLC	15	Activated [50, 51]
TP53	p.Tyr205Asp	193	NSCLC	22	Deleterious [40]

Synonymous variants, common polymorphisms (*KDR* p.Q472H, *KIT* p.M541L, *TP53* p.P72R, *HNF1A* p.G226A) as well as presumably germline variants (*ATM* p.F858L) are not shown. Frameshift and nonsense variants were accounted as deleterious

For variant annotation, the following references were used: ABL1—NP_005148.2; AKT1—NP_005154.2; ATM—NP_000042.3; BRAF—NP_004324.2; CTNNB1—NP_001091679.1; EGFR—NP_005219.2; HNF1A—NP_000536.5; HRAS—NP_001123914.1; KDR—NP_002244.1; KIT—NP_000213.1; KRAS—NP_004976.2; MLH1—NP_000240.1; NOTCH1—NP_060087.3; PIK3CA—NP_006209.2; RB1—NP_000312.2; STK11—NP_000446.1; TP53—NP_000537.3; VHL—NP_000542.1

*LUAD* lung adenocarcinoma, *LUSC* squamous cell lung carcinoma, *NSCLC* non-small cell lung cancer of unknown or mixed histology

### Standard analytic pipelines as a source of possible false positive and false negative findings

Importantly, two Sanger-confirmed EGFR mutations were identified by NGS sequencing, but missed by SVC that is built into the MiSeq Reporter v1.3 + software available in Ilumina maintained BaseSpace™. In particular, inframe deletion in exon 19 that was compounded with single nucleotide variant, a complex mutation p.Glu746_Ser752delinsAlaPhe was mislabeled as two frameshift mutations p.Gly746fs and p.Ser752fs. Though these two molecular changes were found within the same haplotype and, therefore, should be designed as complex mutation, SVC provided no information on this haplotype. Since SVC-reported frameshift deletion lead to total inactivation of the protein rather than activated EGFR that is generated by Sanger-confirmed inframe deletion in exon 19, incorrected calling resulted in a false-negative result. In clinical setting, this false positive would lead to missed opportunity for EGFR TKI treatment. On the other hand, this mutation was successfully detected using Bowtie2 in conjunction with Strelka, Varscan2 and Scalpel pipelines.

Another example of the false negative result was a previously described [52] insertion in exon 19 (p.Ile744_Lys745insLysIleProValAlaIle) that is located 138 bp away from the end of the amplicon. Therefore, at final cycles, reverse reads end within the insertion sequence. Moreover, 3′ end of insertion sequence is similar to the reference sequence. Thus, when BWA [53] or Bowtie2 are run with the default parameters, the reverse reads got misaligned and incorrectly confirm another, previously undescribed shorter insertion. The presence of two insertions in both strand was not reported due to strand bias, and, as a consequence, this mutation was not reported by any tested variant caller, including Strelka, Varscan2 and Scalpel and also Illumina Somatic Variant Caller. However, after adjusting alignment gap open penalty to high and gap extension penalty to low, which resulted in proper alignment, this mutation was successfully identified. Interestingly, realignment with GATK and its standard parameters also allowed successful detection of this mutation, indicating that this aligner should be recommended either as a primary alignment tool or as a backup for independent validation of the findings.

In one patient, the sequencing of *EGFR* amplicons resulted in calling of both p.G719V (or p.Gly719Val) mutation and p.Leu718fs deletion that were present within the same copy of the gene. According to NGS results, all the sequencing reads with mutation p.G719V also had mutation p.Leu718fs, while all other reads from the same amplicon were mutation-free. While the mutation in codon 719 confers the response to EGFR TKI, the frameshift deletion in the preceding codon results in altered protein sequence and premature stop. Therefore, even though p.G719V mutation is present in the genome, it does not express at the level of the protein, and, therefore, the therapy with EGFR TKIs would not be effective. Thus, the detection of p.G719V is, indeed, a false positive, despite its proper identification. Manual analysis of the reads allowed the phasing of p.Leu718fs into the same haplotype, and the correct assessment of the combined effect of both molecular changes.

### The Detection of Somatic Copy Number Variations

Even in absence of the sequenced control samples, the Copy Number Variations (CNVs) may be identified by employing bootstrapping that generates control sets by randomly subsampling experimental ones with replacement. Using this approach, in two specimens an amplification of *EGFR* was detected, with respective increase in its copy number by 5.7 and 17.6 folds. In both cases, this molecular event was detected across all 8 TSACP amplicons. Though *EGFR* amplification does not yield consistent predictive power as previously been shown [54–56], it still may serve as important prognostic marker [57]. In another patient, a significant increase in *MET* gene coverage across its five TSACP amplicons was detected. Typically, the amplification of *MET* is defined by Fluorescent In Situ Hybridization (FISH) as MET:CEP7 ratio that is greater than 2.2. This and higher degrees of amplifications are associated with either primary or acquired resistance to the therapy with EGFR TKIs as well as with enhanced tumorigenesis, invasion and metastasis [58–61]. Moreover, high levels of *MET* amplification defined as MET:CEP7 > 5 were previously identified as actionable driver mutations predicting sensitivity of the tumor to the inhibitors of kinase MET, including crizotinib, currently approved for the treatment of ALK or ROS1-rearranged lung cancers [62]. To investigate the levels of MET amplification in specimen of interest, we normalized the coverage by combining amplicons covering three other genes located on chromosome 7, EGFR, BRAF and SMO. The level of presumable amplification that we observed were low, at 1.65× before normalization and at 1.6× after normalization, thus, indicating that that, in specimen of study, amplification of MET had not reached clinical significance [63].

## Discussion

Advances in target therapy development have introduced an opportunity of informed management of the malignant disorders using the guidance of their molecular profiles. Most conveniently, NGS techniques are capable of the detection of multiple genetic alterations at once, while retaining both an accuracy, informativeness and cost-efficiency. Despite the advantages that NGS techniques provides over conventional methods, their implementation often meets difficulties and should be accompanied equivalency of its analytical performance. For numerous targeted panels, a number of published NGS protocols have already demonstrated satisfactory results. Nevertheless, some of the obstacles remain.

In this study, TSACP technology was tested in the retrospective analysis of somatic mutations in 26 FFPE specimens collected from NSCLC patients. All hotspot alterations detected in clinically relevant genes, including *EGFR, PIK3CA, BRAF, KRAS, NRAS, ERBB2, ALK* and *MET*, were successfully validated by Sanger sequencing or Real-Time PCR. EGFR mutations were detected in eight patients out of 25, while high-prevalent mutations of KRAS were detected in two, and low prevalent in additional three samples. Some findings were less than typical. For example, one of the specimens harbored both activating EGFR p.L858R mutation and PIK3CA p.Glu545Lys mutation that was present at allele frequency of 15%. Another specimen with the deletion of EGFR exon 19 also harbored KRAS p.Gly12Val mutation that was detected at mutant allele frequency of 6%. Though cases of concomitant presence of EGFR and KRAS or PIK3CA mutations have already been described, they are very rare. In both cases, compound mutations were below the limits for the detection by Sanger sequencing [38]. Importantly, the shortcomings of Sanger sequencing and other conventional methods influence historic accumulation of the data on cancer related variants with lower prevalence and, therefore, provide a ground for underrating of these, potentially important, findings. Widespread use of high-sensitive mutation detection techniques should be accompanied by appropriate data analysis and their deposition into panel-specific databases that may facilitate further insights on co-occurrences of various mutations, their allelic frequencies and clinical significance of each finding. The latter aspect remains the most challenging due to common underreporting of the minor alleles.

Clear clinical need of identification of the low-prevalent mutations remains, at least in part, unmet due to use of FFPE for the tumor specimen long-term preservation. FFPE is convenient, cost-effective, and efficient for immunohistochemical staining and morphology analyses. In the meantime, it is unsuitable for the high resolution mutation analysis due to DNA damage artifacts introduced by fixation and paraffin-embedding procedures. In this study, the tissues were fixed using uniform protocol implemented at two independent laboratories. These FFPE samples are routinely produced by clinical workflow.

With the average coverage of 2084×, at least in theory, we were capable to detect mutations at allele frequencies of 1%. According to TCGA data, both lung adenocarcinoma and squamous cell lung cancer specimens harbor approximately 9 mutations per Mb, or, on average, 0.36 mutations per 40 kb, or the size of the TSACP panel [64, 65]. Considering that the sequencing efforts are concentrated in areas intrinsically important for tumorigenesis and, therefore, undergoing the selective pressure, one may expect the mutation rates a bit higher. In our experiment, we detected a bit over 500 variants per 40 kb of sequenced DNA in each sample, on average. In both histological types of lung cancer samples described in TCGA, the proportion of C > T substitutions was at approximately 20%, while in experimentally assessed DNA specimens extracted from FFPE blocks the percentage of C > T base substitutions was substantially higher (p < 0.01 for both lung adenocarcinoma or squamous cell carcinoma, by t-test), in one sample reaching 74%. Moreover, a majority of detected variants were present at frequencies of 0–10% (Fig. 1). Hence, we have to conclude that these changes are more likely to represent DNA degradation artifacts rather than to be a consequence of intratumor heterogeneity [66].

As it was already shown previously, in a majority of FFPE specimens, DNA degradation artifacts are seen in the allele frequency range of 0–10% and represented predominantly by C:G > T:A nucleotide substitutions. It is tempting to dismiss these artifacts by applying simple allele frequency threshold. Nevertheless, in our study we observed that some of the specimens produced a number of artifactual findings presenting as high frequency variants. We noted that the processing of these specimens resulted in remarkably low pre-normalization concentrations of libraries. One of these specimens harbored hotspot AKT1 p.E17K (or p.Glu17Lys) variant at allele frequency of 4%, which significance was overshadowed by highly prevalent artifacts. Therefore, limiting the analysis only to known hotspots while assuming that probability of artifactual detection of the molecular lesion within known hotspot is low would not resolve the reporting dilemma.

**Fig. 3:**
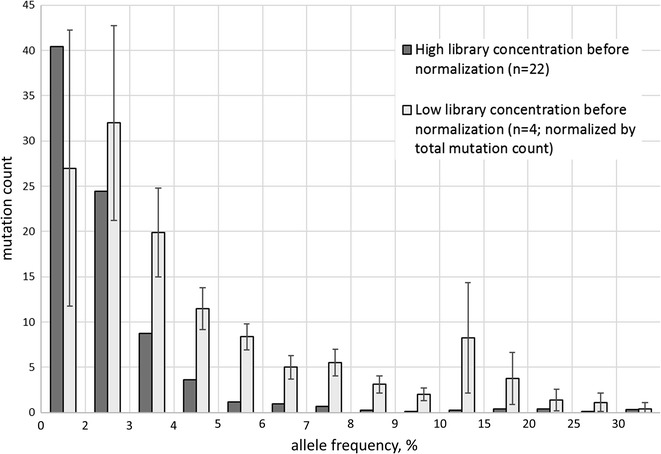
The counts of mutations detected in various ranges of allele frequencies. Samples with pre-normalization library concentrations as low as 1.5 ng/μl were shown to yield substantial amounts of presumably artifactual variants with high prevalence in the sequencing reads. These mutations could not be filtered out by using preset frequency thresholds

In attempt to determine the applicability of using simple mutation allele frequency cut-offs for sorting out FFPE derived artefacts, the relationship of allele frequencies and mutation counts at these frequencies was studied. Figure 3 illustrates that the DNA specimens that resulted in the production of libraries with high pre-normalization concentrations demonstrate a steady decrease in respective mutation counts along with growing frequencies of individual alleles. In contrast, the sequencing of DNA specimens that resulted in the production of libraries with low pre-normalization concentrations identified subset of called variants, with allele frequencies ranging between 9 and 20% (p < 4e−3). This subset could not be clearly filtered by recommended reportable allelic frequency ranges for an analysis of FFPE derived DNA specimens. Thus, it is more difficult to deal with, as it could be identified only when taking into account the range of pre-normalization concentrations for libraries entered into the sequencing run.

In this situation, an assessment of pre-normalization concentrations of libraries may be part for the sample-specific calculation of the detection cut-offs. Moreover, these cut-offs may be even mutation-specific: if the frequency of allele in question is below 10%, but its nature is different from C > T, the probabilities that the variant originated in damaged DNA base would be relatively low. On the other hand, there is some evidence that the DNA degradation artifacts are not exclusively represented by substitutions C:G > T:A. For example, when we applied Fisher’s exact test to mutation counts for all nucleotide substitutions detected in our samples and their frequency ranges, we observed that A > T, T > C, G > T and G > C substitutions may also be associated with increased probability of being FFPE derived artefacts, while for G > C and T > C substitutions these probabilities were significantly decreased (data not shown).

This means that instead of simple empirical rules based on allele frequency cut-offs or nucleotide substitution types, this task may require supervised machine learning approaches executed on paired tumor samples obtained from the same patient and validated on the similar datasets. In any case, the necessity of further research in this field is clear since formalin fixation remains the most common technique for long-term preservation of cancer specimens. An alternative is in introduction of additional library preparation steps that may reduce the counts of FFPE-related artifacts, for example, by treating samples with uracil-DNA glycosylase [16]. It should be also noted that an introduction of additional sample processing steps may influence sequencing outputs, the interpretation of these outputs, and, therefore, clinical decision. In particular, the treatment of samples with uracil-DNA glycosylase may significantly impact mutation allele frequencies [67], which, in turn, are paramount for evaluating potential benefits from the targeted therapy [68]. Therefore, an introduction of additional enzymatic processing requires a comparative study and a through validation before its adoption into the routine.

Despite relatively small size of the study, a single false positive and two false negative calls of the EGFR mutations were uncovered. Importantly, using of two or more combinations of aligning software and further indel realignment with variant callers may help to solve these discrepancies. It is important to note that, for the specimens of question, using of unified and, therefore, simplified analysis protocols would definitely result in non-optimal management of the disease. Aiming at eventual replacement of conventional molecular diagnostics with high-throughput NGS-based methods of NGS, we should remember that commonly used analytic pipelines may remain inefficient when dealing with insertions and deletions, thus, justifying the need for in-house pipelines and scripts.

In addition to the mutation in known hotspots, a total of 24 unique somatic mutations were detected in 26 studied samples. *TP53* was the most mutated gene with a total of 11 alterations detected in 11 patients. Assuming that these non-hotspot mutations were never prospectively validated in NSCLC, and, therefore, never received a designation of actionable item, all of these would have to be classified as incidental findings. Among these were the molecular changes validated as actionable in other types of the tumors, or the mutations that influence the prognosis but not yet targeted by approved medicines and non-hotspot mutations associated with treatment resistance. It seems that the validation of these mutations as actionable may be hastened by deposition of these, not-yet-actionable findings into panel-specific databases that may mined for further insights into their clinical significance.

In addition, a total of five presumably germline coding frame variants were identified, including *KDR* p.Q472H (or p.Gln472His), *KIT* p.M541L (or p.Met541Leu), *TP53* p.P72R (or p.Pro72Arg), *HNF1A* p.G226A (or p.Gly226Ala) and *ATM* p.F858L (or p.Phe858Leu). For a majority of these variants, the frequencies in human populations were high, thus indicating that these variant were likely inherited form the parent. Nevertheless, it may be possible that the presence of one or more of these variants in patient’ genome may have an impact on tumorigenesis or on the therapy outcomes. For instance, the presence of *KDR* p.Q472H variant was previously shown to alter tumor angiogenesis and vascularization [69]. Moreover, in vitro studies showed that the sensitivity to variant-harboring cells to VEGFR2 inhibition is higher than that in the cells with wild-type genotype [69, 70]. As compared to the most common TP53-72P variant, the presence of TP53-72R protein augments apoptosis up to 15-folds, thus, to some degree protecting individual from neoplastic development and, possibly, modifying the response to conventional chemotherapy [71]. Though prognostic value of this polymorphism have already been shown in several case series and prospective studies in several tumor types [72, 73], its predictive role remains not well understood [74–76]. Furthermore, KIT p.M541L variant was reported to confer an enhanced proliferative response to low levels of stem cell factor, though evidences supporting its predictive effect are controversial [77–83]. Finally, despite relatively low frequency of the *ATM* p.F858L variant in human populations described within the 1000 Genomes Project (0.5%), according to previous publications, its nature is likely germline. Interestingly, in large population based cohorts, this variant was associated with an increase in risks for prostate [84], breast [85] and colorectal [86] carcinomas as well as chronic lymphocytic leukemia [87]. Additionally, in patients with childhood T-lineage acute lymphoblastic leukemia, carrying ATM p.F858L was associated with a variety of negative predictors, and worsened outcomes [88].

It is clear that accumulation of the knowledge on the prevalence of these variants in tumor specimens may lead to eventual recognition of these incidental findings as relevant to either personalized selection of therapeutic strategy, or to the risks of the cancer development in the relatives of the proband. No database currently accumulates these kinds of findings in depersonalized form. Therefore, in a majority of the cases, these germline variants remain unreported and, therefore, missed for further analysis. In our opinion, creation of panel-specific incidental finding databases is warranted, as it may hasten overall understanding of tumorigenesis.

## Conclusion

Reporting of entire mutational spectrum revealed by targeted sequencing is questionable, at least until the clinically-driven guidelines on reporting of somatic mutations are established. The need for the development of panel-specific databases allowing analysis of co-occurrence and relevance of somatic mutations, CNVs and germline variants in de-identified form is evident. Further standardization of sequencing protocols, especially their data analysis components, may require assay-, disease-, and, in many cases, even sample-specific customization that could be performed only in cooperation with clinicians.

## Abbreviations

TKI: tyrosine kinase inhibitor
NSCLC: non-small cell lung cancer
PCR: polymerase chain reaction
NGS: Next Generation Sequencing
CLIA: Clinical Laboratory Improvement Amendments
CAP: College of American Pathologists
TSACP: TruSeq Amplicon—Cancer Panel
RCRC: Russian Cancer Research Centre
RSCRR: Russian Scientific Center of Roentgenology and Radiology
FFPE: Formalin Fixed, Parffine Embedded
ASCO: American Society of Clinical Oncology
CDF: cumulative distribution function
SVC: Illumina Somatic Variant Caller (Illumina Inc, San Diego, California)
CNV: Copy Number Variations
FISH: Fluorescent In Situ Hybridization
